# Outbreak of *Dirkmeia churashimaensis* Fungemia in a Neonatal Intensive Care Unit, India

**DOI:** 10.3201/eid2604.190847

**Published:** 2020-04

**Authors:** Anuradha Chowdhary, Kothapalli Sharada, Pradeep Kumar Singh, Dalip Kumar Bhagwani, Nitin Kumar, Theun de Groot, Jacques F. Meis

**Affiliations:** Vallabhbhai Patel Chest Institute, University of Delhi, Delhi, India (A. Chowdhary, P.K. Singh);; Hindu Rao Hospital, Delhi (K. Sharada, D.K. Bhagwani);; Wellcome Trust Sanger Institute, Hinxton, UK (N. Kumar);; Canisius-Wilhelmina Hospital (CWZ), Nijmegen, the Netherlands (T. de Groot, J.F. Meis);; Centre of Expertise in Mycology Radboudumc/CWZ, Nijmegen (J.F. Meis)

**Keywords:** *Dirkmeia churashimaensis*, outbreak, bloodstream infections, neonatal intensive care unit, whole-genome sequencing, antimicrobial resistance, yeast, fungi, India

## Abstract

Bloodstream infections caused by uncommon or novel fungal species are challenging to identify and treat. We report a series of cases of fungemia due to a rare basidiomycete yeast, *Dirkmeia churashimaensis*, in neonatal patients in India. Whole-genome sequence typing demonstrated that the patient isolates were genetically indistinguishable, indicating a single-source infection.

During the past decade, outbreaks of bloodstream infections (BSIs) caused by rare and challenging to identify fungi have increased (*1*). Many rare infections occurred among patients admitted to neonatal intensive care units (NICUs). Rarely, yeasts belonging to the phylum Basidiomycota, including genera *Malassezia, Trichosporon*, and *Rhodotorula* (*2**–**4*), have been implicated in outbreaks in NICUs. 

*Dirkmeia churashimaensis* (previously *Pseudozyma churashimaensis*) is a rare basidiomycete, ustilaginomycetous, anamorphic yeast first isolated in 2008 from the leaves of sugar cane (*Saccharum officinarum*) in Okinawa, Japan (*5*). Initially, *D. churashimaensis* was identified as a novel *Pseudozyma* species on the basis of morphological and physiologic aspects and by molecular analysis of the D1/D2 domains and internal transcribed spacer (ITS) regions (*5*). In 2015, Wang et al. used multigene phylogeny and proposed that *P. churashimaensis* represents a new genus, *Dirkmeia* gen. nov. (*6*). *Dirkmeia* gen. nov. is a common endophytic yeast found in the leaf tissues of rice, corn, sugar cane, and pepper plants (*7*)*.* Of note, foliar application of this leaf-colonizing yeast has been reported to control plant viral disease under field conditions (*8*)*.* However, *D. churashimaensis* has not been reported to cause human infections. We report an unusual cluster of 12 cases of fungemia caused by *D. churashimaensis* among NICU patients in a multispecialty hospital in Delhi, India.

## The Study

During June 2016–January 2017, a total of 12 cases of fungemia occurred among neonates admitted to a 24-bed NICU of a multispecialty hospital in Delhi. Cases of BSI were defined as the isolation of *D*. *churashimaensis* from >1 peripheral blood culture in patients with signs and symptoms of sepsis. The first case of fungal sepsis was observed in a preterm infant with very low birthweight (<1,500 g) who experienced asphyxia during birth. The patient’s blood culture was positive for yeast on day 6 after birth. Fluconazole treatment was initiated by administering a loading dose of 12 mg/kg bodyweight, after which the patient received 6 mg/kg bodyweight in addition to vancomycin and meropenem. The second case occurred 2 weeks later in a preterm baby with low birthweight (<2,500 g) whose blood culture yielded *D. churashimaensis* on day 2. During the next 6 months, 10 additional cases of *D*. *churashimaensis* BSI were identified (Table 1).

**Table 1:** Clinical details of patients with *Dirkmeia churashimaensis* fungemia in a neonatal intensive care unit, India*

Pt no.	Isolate ID	GA, wk/sex	DOB; delivery method	Birthweight, g	Date blood sample collected	Day of positive blood test†	Risk factors	Antifungal therapy‡	NICU stay, d; outcome
1	VPCI 2456/P/16§	31/M	2016 Jun 25; LSCS	1,400	2016 Jun 25	6	PT, VLBW, IUGR, thrombocytopenia, CVC, severe asphyxia, sepsis, mechanical ventilation	FLU, 10 d; VAN and MER, 7 d	15; survived
2	VPCI 2478/P/16	29/M	2016 Jul 7; LSCS	1,100	2016 Jul 8	2	PT, VLBW, IUGR, sepsis, thrombocytopenia	FLU, 14 d; VAN and MER, 10 d	12; survived
3	VPCI 2510/P/16	29/F	2016 Jul 19; VD	1,000	2016 Jul 19	4	PT, VLBW, sepsis, thrombocytopenia	FLU, 14 d; VAN and MER, 14 d	18; survived
4	VPCI 2515/P/16§	27/F	2016 Aug 30; VD	1,000	2016 Aug 31	4	PT, VLBW, IUGR, thrombocytopenia, maternal history of preeclampsia, antepartum hemorrhage	FLU, 14 d; AMI and CIP, 12 d	16; survived
5	VPCI 2601/P/16	32/F	2016 Sep 24; LSCS	1,200	2016 Sep 24	3	PT, VLBW, persistent hypoglycemia, severe asphyxia, sepsis, CVC, mechanical ventilation	FLU, 10 d; VAN and MER, 12 d	6; died
6	VPCI 2634/P/16	27/M	2016 Oct 17; VD	750	2016 Oct 17	5	Extremely PT, ELBW, severe asphyxia, CVC, sepsis, mechanical ventilation	FLU, 10 d	8; died
7	VPCI 2699/P/16§	30/M	2016 Nov 9; VD	800	2016 Nov 9	2	PT, ELBW, sepsis, persistent hypoglycemia, severe asphyxia, CVC, mechanical ventilation	FLU, 10 d; VAN and MER, 10 d	11; died
8	VPCI 2759/P/16	33/M	2016 Nov 26; LSCS	1,200	2016 Nov 28	3	PT, VLBW, severe asphyxia, CVC, thrombocytopenia, persistent hypoglycemia, mechanical ventilation	FLU, 14 d; VAN and MER, 10 d	10; survived
9	VPCI 2801/P/16§	27/M	2016 Dec 14; VD	1,100	2016 Dec 14	4	PT, VLBW, severe asphyxia, thrombocytopenia, sepsis, CVC, mechanical ventilation	FLU, 18 d; VAN and MER, 12 d	24; survived
10	VPCI 2845/P/16	27/F	2016 Dec 28; LSCS	1,200	2016 Dec 28	6	PT, VLBW, thrombocytopenia, mechanical ventilation, sepsis, persistent hypoglycemia, CVC	FLU, 10 d; VAN and MER, 8 d	8; died
11	VPCI 2224/P/17§	30/M	2017 Jan 3; LSCS	1,350	2017 Jan 3	4	PT, VLBW, sepsis, persistent hypoglycemia, CVC	FLU, 10 d; VAN and MER, 10 d	14; survived
12	VPCI 2271/P/17§	29/F	2017 Jan 18; VD	1,000	2017 Jan 19	4	PT, VLBW, thrombocytopenia, mechanical ventilation, CVC, sepsis, persistent hypoglycemia	FLU, 10 d; VAN and MER, 12 d	9; died

*AMI, amoxicillin; CIP, ciprofloxacin; CVC, central venous catheter; DOB, date of birth; ELBW, extremely low birthweight (<1,000 g); FLU, fluconazole; GA, gestational age; ID, identification; IUGR, intrauterine growth restriction; LSCS, lower segment cesarean section; MER, meropenem; PT, preterm; Pt., patient; VAN, vancomycin; VPCI, Vallabhbhai Patel Chest Institute (Delhi, India); VD, vaginal delivery; VLBW, very low birthweight (<1,500 g). †Indicates days after birth. ‡All patients were given a 1-time loading dose of 12 mg/kg bodyweight of FLU and then 6 mg/kg bodyweight. §Isolates selected for whole-genome sequencing.

Isolates grew as yeast-like cream to pale yellow, dry, and wrinkled colonies with fringes on the margin on Sabouraud glucose agar at 35°C after 48 h of incubation; the isolates grew slowly at 37°C over 3 days. Micromorphology showed fusiform yeast cells with hyphae and polar budding with short denticles. Identification by VITEK2 (bioMérieux, https://www.biomerieux.com) yielded *Cryptococcus laurentii* with 88% probability. We conducted carbon assimilation on isolates and noted assimilation of D-trehalose and N-acetyl-glucosamine at 37°C in 48 h.

Because no multilocus sequence or microsatellite typing were available, we used whole-genome sequencing (WGS) and amplified fragment-length polymorphism typing to understand the genetic relationships among isolates. We conducted matrix-assisted laser desorption/ionization-time of flight mass spectrometry by using Biotyper 3.1 (Bruker Corp., https://www.bruker.com) to identify the yeasts. However, because no database of this yeast is available, we were not able to make an identification. The in-house database created yielded correct identification in the remaining 10 isolates with high score values (>2). 

We used isolates ITS and D1/D2 regions to sequence isolates, as described previously (*9*). We searched ITS and D1/D2 region sequences in BLAST (https://blast.ncbi.nlm.nih.gov) and identified isolates from the outbreak as *D. churashimaensis*. The isolates had >99% identity with *D. churashimaensis* sequences from GenBank (accession nos. MN758637–48 and MN158668–79). 

**Figure 1:**
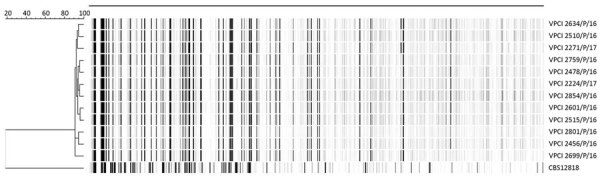
Dendrogram of amplified fragment-length polymorphism analysis of *Dirkmeia churashimaensis* isolated from 12 cases of fungemia in patients in a neonatal intensive care unit, Delhi, India. The dendrogram was constructed by using unweighted pair group method with averages and the Pearson correlation coefficient. Dendrogram was restricted to fragments of 60–400 bp. CBS12818, a *Pseudozyma aphidis* isolate previously reported from neonatal fungemia in India, was included in the analysis. Scale bar indicates the percentage similarity. VPCI, Vallabhbhai Patel Chest Institute (Delhi, India).

We used MEGA 7 (https://www.megasoftware.net) to perform phylogenetic analysis of ITS sequences by using the neighbor-joining method with 2,000 bootstrap values. All isolates from the outbreak clustered together. The reference *D. churashimaensis* isolate formed a distinct cluster but showed 99% nucleotide similarity with isolates from the outbreak. We performed amplified fragment-length polymorphism fingerprint analysis, as described previously (*10*), which yielded identical banding pattern among all 12 isolates, suggesting clonal origin (Figure 1).

**Figure 2:**
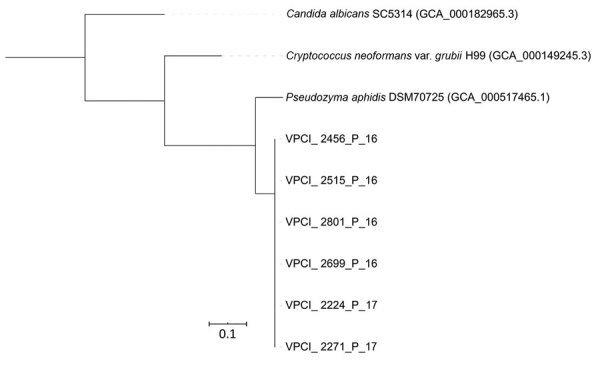
Whole-genome single-nucleotide polymorphism–based phylogenetic tree of 6 *Dirkmeia churashimaensis* isolates from cases of fungemia among patients in a neonatal intensive care unit, India. Other yeast species included for comparison. Scale bar indicates single-nucleotide polymorphism differences per site. VPCI, Vallabhbhai Patel Chest Institute (Delhi, India).

We performed WGS on 6 isolates by using IonPGM (IonTorrent; ThermoFisher, https://www.thermofisher.com) next-generation sequencing technology, following the manufacturer’s protocol. We deposited sequences into BioProject (accession no. PRJEB35981). We identified average nucleotide identity and SNPs by comparing 6 genomes in MUMmer (http://mummer.sourceforge.net) and compared all the genomes against other publicly available basidiomycete yeast genomes (Table 2) by using progressiveMauve (*11*). We constructed a whole-genome SNP-based phylogenetic tree by using SplitsTree4 (*12*; Figure 2). The assembled genome size of *D. churashimaensis* is ≈21 Mb with a G+C content of 58%. The assembly contained 397–502 contigs ranging in length from 535,979 to 946,852 bp (average contig length 741,415 bp). *D. churashimaensis* isolates were genotypically indistinguishable and had 99.6% similarity among the genomes (average nucleotide identity >99.6%; Table 2). The average number of SNP differences between isolates was 1,074.5 (range 402–1,621), indicating high clonality.

**Table 2:** Results of average nucleotide identity analysis giving percentage similarity among *Dirkmeia churashimaensis* isolates from patients in a neonatal intensive care unit, India, compared with other basidiomycetes and *Saccharomyces cerevisiae* isolates*

Isolate ID	Isolate ID					
	VPCI 2456/P/16	VPCI 2515/P/16	VPCI 2699/P/16	VPCI 2801/P/16	VPCI 2224/P/17	VPCI 2217/P/17
VPCI 2456/P/16	100	99.86	99.86	99.87	99.86	99.86
VPCI 2515/P/16	99.87	100	99.89	99.91	99.93	99.91
VPCI 2699/P/16	99.86	99.9	100	99.92	99.9	99.91
VPCI 2801/P/16	99.88	99.91	99.92	100	99.89	99.9
VPCI 2224/P/17	99.89	99.91	99.91	99.92	100	99.89
VPCI 2271/P/17	99.88	99.87	99.88	99.89	99.89	100
*Cutaneotrichosporon oleaginosus*	68.22	68.82	68.51	68.68	68.54	68.57
*Cryptococcus neoformans*	70.94	69.27	69.99	70.37	70.15	69.95
*Trichosporon asahii*	69.54	63.37	69.32	69.66	69.78	69.88
*Saccharomyces cerevisiae*	64.82	63.40	63.56	64.24	64.48	64.52

*ID, identification; VPCI, Vallabhbhai Patel Chest Institute (Delhi, India).

We conducted antifungal susceptibility testing using microbroth dilution method published by the US Clinical Laboratory Standards Institute (*13*). As expected with Basidiomycota, isolates in this outbreak were resistant to echinocandins. Susceptibility testing showed that all isolates were resistant to caspofungin, anidulafungin, and micafungin (MICs >8 µg/mL). However, all isolates had low MICs for azoles, including voriconazole (MIC 0.03–0.125 µg/mL; geometric mean [GM] 0.04 µg/mL), isavuconazole (MIC 0.03–0.125 µg/mL; GM 0.05 µg/mL), itraconazole (MIC 0.03–0.25 µg/mL; GM 0.057 µg/mL), posaconazole (MIC 0.03–0.25 µg/mL; GM 0.092 µg/mL), and fluconazole (MIC 1–4 µg/mL; GM 2.37 µg/mL). Amphotericin B (GM MIC 0.198 µg/mL) and 5-flucytosine (GM MIC0.157 µg/mL) had potent activity. 

All patients were treated with fluconazole at a loading dose of 12 mg/kg bodyweight and then 6 mg/kg for 10–14 days; 5 patients died, a case-fatality rate of 42%. All patients had risk factors, such as preterm birth or low or very low birthweight, and 8/12 were intubated (Table 1). The most serious risk factors were central venous catheter (n = 9), thrombocytopenia (n = 8), and severe asphyxia (n = 6). The age at the onset of fungemia ranged from 2 to 6 days, and the attack rate was 0.33 during the 6-month outbreak. The mean gestational age was 29.2 weeks and the mean birthweight was 1.1 kg. Altogether, 11 patients had 1–6 days of antimicrobial drug therapy before isolation of yeast in blood culture. 

After the second case of fungemia was identified, infection control measures were implemented and surveillance cultures obtained to trace the source of infection. Doctors, nursing staff, and assistants in the NICU were screened for hand carriage of the yeast, and extensive sampling of fomites including floors, equipment, disinfectants, vials, and infusion pumps was conducted. All environmental cultures were negative, and no other cases of fungemia due to *D*. *churashimaensis* were identified after continued compliance with infection control measures, including rigorous handwashing practice.

## Conclusions

Our report highlights not only clinical importance of rare yeast species in the NICU but also emphasizes that WGS provides a highly sensitive tool for genotyping pathogens without prior knowledge of the genomes (*1*,*14*,*15*). Major healthcare-associated outbreaks of uncommon and novel fungal species have occurred in recent years, including *Candida auris* from a clonal outbreak in India (*9*). Mycologists should be vigilant when they isolate unusual or rare yeasts with potential antifungal resistance. Considerable challenges remain in the diagnosis of rare and unusual yeasts and the risk for misidentification of cases and outbreaks is high. Our findings reinforce the need for awareness of this new fungal risk among the public health community.
